# Report on Morton's Claim of the Discovery of Anæsthetic Agents

**Published:** 1853-01

**Authors:** 


*Report on Morton's Claim of the Discovery of Anæsthetic Agents.* House of Representatives, 1852.

This is a voluminous account of the ether controversy, and seems to be the result of careful and dispassionate examination. The result is known to our readers long since through the papers. Morton's claim is there fully sustained.

